# Transcranial magnetic stimulation to probe the role of the supplementary motor area in tics

**DOI:** 10.3389/fnhum.2025.1720968

**Published:** 2025-12-04

**Authors:** Christine A. Conelea, Brianna C. M. Wellen, Sunday M. Francis, Bryon A. Mueller, Suma Jacob, Kelvin Lim, Benjamin D. Greenberg

**Affiliations:** 1 Department of Psychiatry and Behavioral Sciences, University of Minnesota, Minneapolis, MN, United States; 2 Butler Hospital, Center for Neurorestoration and Neurotechnology, Providence VA Medical Center, Brown University, Providence, RI, United States

**Keywords:** tic, Tourette, child, brain stimulation, transcranial magnetic stimulation, supplementary motor area

## Abstract

**Introduction:**

Supplementary motor area (SMA) hyperactivity is thought to be a key neural mechanism in tics. This study probed SMA’s role in tic expression, voluntary tic control, and premonitory urge experiences using one session of 1 Hz “inhibitory” repetitive transcranial magnetic stimulation (rTMS) targeting SMA in a repeated measures, small-N experimental design.

**Methods:**

Youth with Tourette Syndrome (TS) ages 12—17 years (*N* = 14) completed a clinical assessment and MRI to localize SMA. The video-based Tic Suppression Task (TST) quantified tic frequency and urges during conditions of Free-to-Tic, Suppression, and Suppression+Reward. The TST was followed by randomly assigned active 1 Hz (*n* = 8) or sham rTMS (*n* = 6) and TST repetition post-stimulation.

**Results:**

Active rTMS led to greater tic frequency reductions during Free-to-Tic (*d* = 0.34) and Suppression+Reward (*d* = 0.24) but not Suppression (*d* = 0.0). A stronger effect size for active rTMS was observed in both suppression conditions (*d* = 0.26, *d* = 0.63) when excluding participants classified as baseline “strong suppressors” (*n* = 5). Urges did not differ group-wise for Free-to-Tic (*d* = 0.09) but decreased more following active rTMS in both suppression conditions (*d* = 0.19, *d* = 0.52).

**Discussion:**

Overall, results suggest that the acute aftereffects of active 1 Hz rTMS to SMA may include reduced natural tic frequency, improved tic controllability, and lower urge intensity, especially while engaged in suppression efforts. Results are consistent with prior literature pointing to SMA hyperactivation in TS and suggests the potential therapeutic value of rTMS.

## 1 Introduction

Tics are rapid, repetitive, involuntary motor movements and vocalizations often preceded by aversive somatic sensations called “premonitory urges.” Tics are the hallmark symptom of Tourette Syndrome (TS), which is associated with impairments across multiple functional domains (Conelea et al., 2013) and highly co-occurs with psychiatric disorders, particularly those with overlapping corticostriatal circuitry dysfunction [e.g., Attention Deficit Hyperactivity Disorder, Obsessive Compulsive Disorder; (Freeman et al., 2000; Wang et al., 2011)].

Tic reduction is the primary clinical target of existing evidence-based treatments, including medications (e.g., α-agonists, antipsychotics) and Comprehensive Behavioral Intervention for Tics (CBIT), which teaches behavioral skills to enhance voluntary tic control. About 40%–50% of patients experience clinically significant improvement with these interventions (Pringsheim et al., 2019). Refined understanding of the neural mechanisms underlying tic expression and inhibition is needed to develop targeted, individualized treatments that may increase response rates.

Converging evidence indicates that tics and related premonitory urges result from aberrant activity in parallel, integrated cortico-striatal-thalamo-cortical circuits (CSTC); (Mink, 2006; Wang et al., 2011), including (a) excessive activity in dorsolateral striatum (DLS, aka putamen)-sensorimotor cortex circuitry involved in movement output and (b) diminished activity in dorsomedial striatum (DMS, aka caudate)-prefrontal cortex circuitry underlying cognitive control (Wang et al., 2011). Premonitory urges are thought to arise from increased activity in DLS-driven sensorimotor circuitry in conjunction with limbic and paralimbic areas, such as the amygdala, insula, and mid-cingulate (Yael et al., 2015).

The supplementary motor area (SMA) is a cortical node within DLS-CSTC pathways. SMA receives projections from prefrontal cortex and motor areas of the cingulate cortex and projects directly to the primary motor cortex (Tanji, 1994). SMA links contextual cues to motor actions and plays a strong role in motor inhibition via direct projections to the striatum and a “hyperdirect” connection to the subthalamic nucleus (Nachev et al., 2008).

Supplementary motor area dysfunction, namely hyperactivity and hyperconnectivity, is thought to be a key neural mechanism involved in tics. TS patients show enhanced functional (Wang et al., 2011) and structural (Worbe et al., 2015) connectivity between SMA and successive nodes of the CSTC sensorimotor circuit, including primary motor cortex (Hampson et al., 2009). Decreased concentrations of the inhibitory neurotransmitter GABA in SMA are associated with more severe and frequent premonitory urges (He et al., 2022). Tic severity and complexity (Worbe et al., 2012) and premonitory urge severity (Zapparoli et al., 2015) are correlated with SMA activation and functional connectivity abnormalities, and SMA activity is abnormally elevated prior to tic execution (Bohlhalter et al., 2006; Hampson et al., 2009; Wang et al., 2011) and during periods of higher tic frequency (Hampson et al., 2009; Stern et al., 2000). SMA shows strong resting state functional connectivity with deep brain stimulation sites most effective for treating tics (Fox et al., 2014) and with striatal regions thought to change with CBIT treatment (Deckersbach et al., 2014).

Brain stimulation studies probing the functional role of SMA are also suggestive of its involvement in TS. Direct stimulation of SMA with subdural electrodes produces urges to move in patients with epilepsy (Fried et al., 1991). Tic-like movements, echophenomena, and urges to move have been induced in healthy participants using excitatory repetitive transcranial magnetic stimulation (rTMS; (Finis et al., 2013)). In patients with TS, inhibitory stimulation of SMA using 1 Hz rTMS has been associated with reduced tic severity in case reports (Mantovani et al., 2007) and open label trials (Kahl et al., 2021a; Kwon et al., 2011; Le et al., 2013; Mantovani et al., 2006). Small, randomized, sham-controlled trials targeting SMA inhibition with 1 Hz rTMS (Landeros-Weisenberger et al., 2015), deep TMS with the HBDL coil (Bloch et al., 2016), and continuous theta burst stimulation (cTBS) (Wu et al., 2014) did not find group-level clinically meaningful change, although these were generally brief trials and an extended open-label 1 Hz rTMS trial (daily for 6 weeks) did improve outcomes (Landeros-Weisenberger et al., 2015). Our understanding of the neural correlates involved in these rTMS treatments is limited to a single study that showed decreased SMA activation and connectivity to bilateral primary motor cortex during an fMRI finger tapping task following a 2 days cTBS regimen (Wu et al., 2014).

Taken together, the converging evidence regarding CSTC neurocircuitry abnormalities in TS suggests that SMA is a critical cortical node for tic generation, voluntary tic control, and premonitory urge experiences. The primary aim of the current study was to directly probe the functional role of SMA in natural tic expression, voluntary tic control, and premonitory urge intensity using a single session of rTMS to acutely induce change in SMA function. We hypothesized that inhibition of SMA using inhibitory, low-frequency 1 Hz rTMS would lead to reduced tic frequencies during periods of both tic expression and attempted suppression, and that it would also lead to lower premonitory urges.

## 2 Materials and methods

### 2.1 Participants

Participants were *N* = 14 youth ages 12–17 years who met DSM-5 criteria for TS. This age range was selected based on safety considerations for non-therapeutic rTMS (vs. younger patients) and because this tends to be the developmental period of peak tic severity and related social distress [vs. adults; (Steinberg et al., 2010)]. Participants were recruited via advertising, flyers, and distribution of study information to local healthcare providers. The study was posted on ClinicalTrials.gov (NCT02205918). This project was first approved by the Institutional Review Boards at Rhode Island Hospital/Lifespan, Butler Hospital, and later at the University of Minnesota (due to PI relocation). Consent was obtained from parents/guardians and assent from children prior to data collection. Participant characteristics are presented in Table 1.

**TABLE 1:** Sample demographics, clinical characteristics, and baseline Tic Suppression Task data.

Characteristic	Active (*n* = 8)	Sham (*n* = 6)
**Demographics**		
Sex (male)	4 (50%)	6 (100%)
Age (years)	14.63 (1.77), 12–17	13.83 (1.47), 12–16
Medicated (yes)	6 (75%)	4 (80%)*
ADHD	6	2
Anxiety	6	3
OCD	1	1
Major depressive disorder	1	0
**Clinical measures**		
**Measure**	**Active mean (SD)**	**Sham mean (SD)**
YGTSS motor	14.63 (4.57)	10.83 (3.32)
YGTSS vocal	10.38 (6.05)	8.33 (5.16)
YGTSS total	25.00 (10.13)	19.00 (6.20)
PUTS total	17.86 (5.46)	21.00 (5.06)
**Tic suppression task: average tics per minute**		
**Condition**	**Active mean (SD), range**	**Sham mean (SD), range**
Pre-TMS FT	15.30 (13.51), 3.04–33.73	9.97 (9.92), 3.54–29.83
Post-TMS FT	5.35 (5.44), 0.69–13.60	3.98 (4.57), 0.33–10.24
Pre-TMS SUP	3.73 (3.77), 0.33–10.58	3.61 (2.45), 0.16–6.55
Post-TMS SUP	2.76 (3.48), 0.42–9.97	2.64 (1.90), 0.17–5.87
Pre-TMS SUP+R	3.14 (5.17), 0.00–15.06	1.35 (1.24), 0.00–3.65
Post-TMS SUP+R	2.26 (3.61), 0.00–10.09	0.83 (0.77), 0.00–2.18
**Tic suppression task: average urge rating**		
**Condition**	**Active mean (SD), range**	**Sham mean (SD), range**
Pre-TMS FT	3.17 (0.94), 2.03–4.67	2.26 (1.52), 0.89–4.00
Post-TMS FT	1.73 (1.15), 0.45–3.78	1.05 (0.78), 0.00–2.10
Pre-TMS SUP	3.98 (2.32), 0.00–7.60	3.16 (1.78), 1.33–5.80
Post-TMS SUP	2.36 (1.06), 0.80–4.10	2.04 (2.03), 0.00–5.30
Pre-TMS SUP+R	3.63 (1.83), 1.40–6.38	3.90 (1.51), 2.18–5.90
Post-TMS SUP+R	1.63 (1.22), 0.00–3.17	2.76 (1.64), 0.96–5.30

*Significant difference between groups at baseline (independent-samples *t*-test).

#### 2.1.1 Inclusion/exclusion criteria

To be included, the participants’ tics must have occurred at a rate of ≥1 observable tics per minute, a criterion used previously to enable detection of potential changes in tic frequency (Conelea et al., 2011). Exclusion criteria were: (1) medical conditions associated with altered TMS risk profile (e.g., known intracranial pathology, epilepsy or seizure disorder, traumatic brain injury, brain tumor, stroke, implanted medical devices, pregnancy), (2) inability to undergo MRI, (3) left handedness, (4) previous diagnosis of psychosis, autism spectrum disorder, or cognitive disability, (5) substance abuse or dependence within the past year, (6) current suicidal intent, (7) history of ≥3 sessions of CBIT, and (8) current neuroleptic medications (given concerns around lowered seizure threshold). Other tic or psychiatric medications were allowed provided they remained stable throughout the study period. A study physician reviewed assessment information and relevant medical records as part of eligibility determination.

### 2.2 Assessments

Demographic information was collected to characterize the sample. Tic symptoms were rated using the clinician-administered Yale Global Tic Severity Scale (Leckman et al., 1989), and psychiatric comorbidities were assessed using the Mini International Neuropsychiatric Interview [MINI-KID (Sheehan et al., 2010)]. A 5 min videotaped observation, in which the child was recorded while alone in a room, was used to establish the tic frequency inclusion criterion and inform operational definitions of tics for coding.

### 2.3 MRI

Structural and functional MRI data were collected on a Siemens PRISMA 3T scanner to locate SMA within-participant, following procedures used in the Wu et al. (2014) prior TMS-SMA study in TS and established methods for deriving task-related functional activation maps of the primary motor cortex and SMA (Witt et al., 2008). A high-resolution T1-weighted MPRAGE of the entire brain was acquired in the sagittal plane for anatomical reference (256 × 256 matrix, field of view = 256 mm^2^, voxel size = 1.0 mm × 1.0 mm × 1.0 mm). For SMA functional localization, fMRI EPI images were acquired during a bilateral finger-thumb tapping task [band = 4, TR = 1,000 ms, TE = 30 ms, field of view = 192 mm^2^, slice thickness = 2 mm (60 slices)]. Images were preprocessed in AFNI using afni_proc.py (AFNI, 2014) to generate participant-specific BOLD motor activation maps contrasting blocks of tapping versus rest.

### 2.4 Experimental design

**FIGURE 1:**
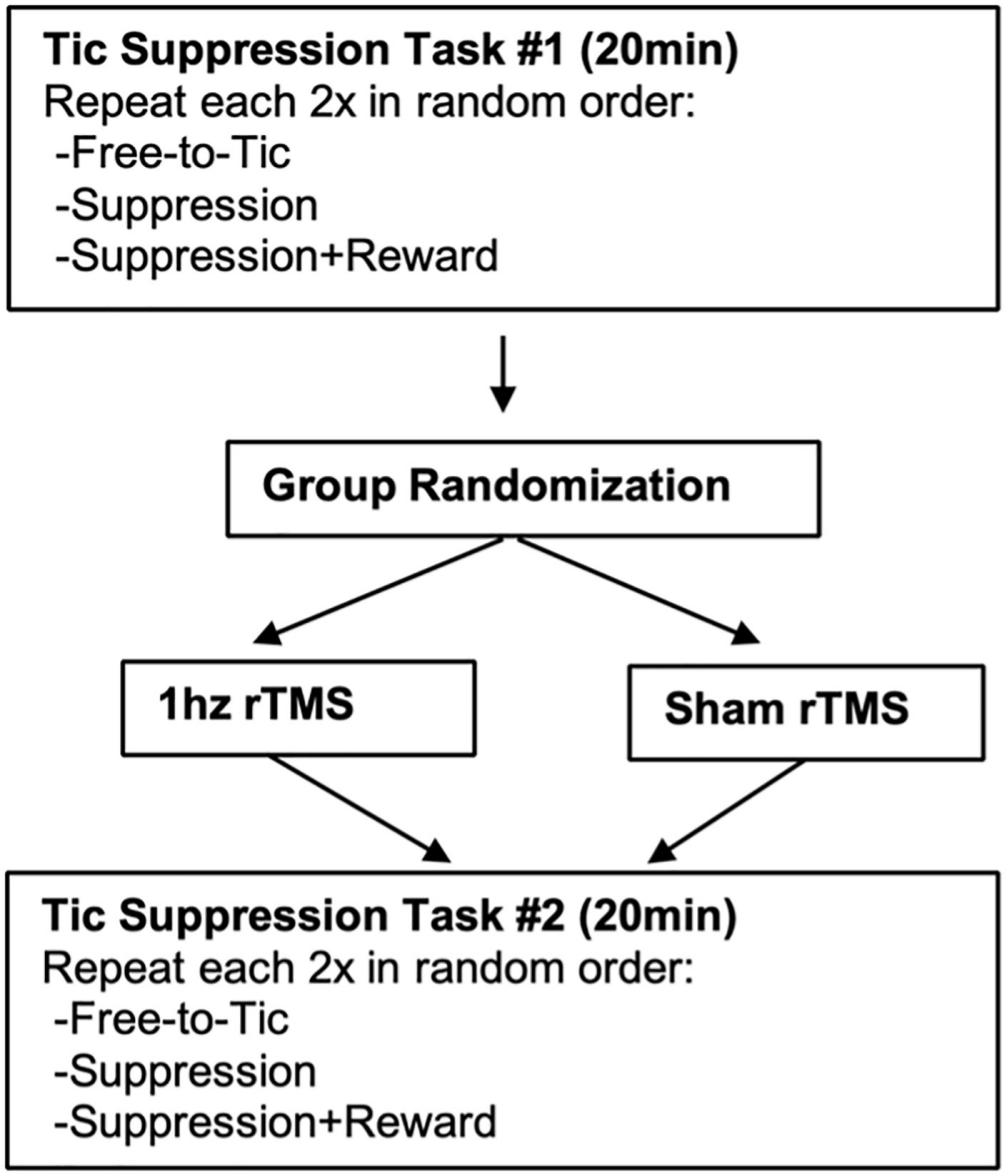
Study design.

The study used a randomized, sham-controlled, repeated measures, small-N experimental design, an approach that enables testing with a smaller number of participants (Krasny-Pacini and Evans, 2018). Two study visits occurred within seven calendar days (visit 1: assessment and brain MRI; visit 2: rTMS and Tic Suppression Task, TST). The experimental procedure sequence is illustrated in Figure 1. All participants first completed the TST, described below. Participants were randomized to then receive either active 1 Hz (*n* = 8) or sham rTMS (*n* = 6) using an urn randomization procedure blocking on gender, age (12–14 vs. 15–18), and tic severity [YGTSS Total < 22 vs. ≥ 22, where 22 is the reported mean score in tic samples (Leckman et al., 1989)]. Participants then repeated the TST, allowing for examination of potential changes in tic frequencies and premonitory urge intensity due to rTMS.

### 2.5 Tic Suppression Task (TST)

In this established paradigm (Conelea et al., 2018; Himle and Woods, 2005), youth are video-recorded while alone in a room in front of a computer. Participants were told that the computer was a “tic detector” that could deliver points exchangeable for a small amount of money. In reality, participants were observed via video feed, and all youth received an equivalent gift card. This deception was designed to ensure that points functioned as reinforcers and to minimize reactivity of tics to observation (Piacentini et al., 2006). Parents were informed of this procedure during the consent process, and debriefing and assent with the child occurred at the end of their last study visit.

Participants were exposed to three, 3 min conditions: (1) Free-to-Tic (FT), in which the participant was asked to simply sit and tic naturally, (2) Suppression (SUP), which instructed the participant to suppress tics (“Do whatever you need to do while staying seated to keep your tics from happening”), and (3) Suppression plus Reward (SUP+R), which included SUP instructions plus delivery of a visual “point” for each 10 s tic-free interval (“For every 10 s without a tic, the tic detector will give you a point”). This rewards schedule has been shown to enhance tic control efforts (Capriotti et al., 2017) and was included to mimic contexts in which reinforcers for suppression are present (e.g., skills practice during CBIT therapy). Conditions were randomly repeated two times each.

Premonitory urge intensity was captured during all TST conditions using a well-established method (Himle et al., 2007; Woods et al., 2009). Every 30 s, a visual scale appeared on the computer, prompting participants to verbally rate urge intensity on a scale of 0–8. Participants were instructed in the use of scale and its anchors prior to the first TST administration.

Tic Suppression Task video recordings were later coded to establish tic frequencies and record urge ratings during each condition using computerized behavioral coding (Datavyu Team, 2014). Coders were masked to rTMS status, but removing all cues about condition instructions was infeasible given the need to see and hear participants to code. Tic frequency scores for each condition were calculated by summing the number of tics occurring during the condition and dividing it by three, for a mean tic per minute score. Urge ratings were converted to an average for each condition. An independent rater coded 20% of videos to establish interrater reliability (*k* ≥ 0.8).

### 2.6 rTMS procedures

Transcranial magnetic stimulation was delivered using a Magstim Super Rapid2 stimulator (Magstim Company Ltd., United Kingdom). Resting motor threshold (RMT) was identified using single-pulse TMS administered to the contralateral hand area of the primary motor cortex prior to rTMS administration. RMT was defined as the minimum intensity needed to elicit a threshold EMG response (50 mV in peak-to-peak amplitude) in a resting target muscle (abductor pollicis brevis) in 5/10 trials. For rTMS, coil placement was guided by a neuronavigation platform (BrainSight 2.2.7, Rogue Research, Montreal, Quebec, Canada) using the participant’s individual anatomical MRI and motor task activation maps; reconstructions in BrainSight were visually inspected by a study physician to locate an area of peak motor task activation within the SMA region. Neuronavigation remained active throughout the entire rTMS session to provide continuous positional verification, and the coil operator actively adjusted the coil as needed to remain on target when participant movements occurred. For all participants, the coil handle was oriented towards the occiput, centered over the midsagittal plane. Stimulation was delivered at 1 Hz in a single 33-min train (2,000 pulses) at 110% of RMT. Active stimulation used a vacuum-cooled 70-mm figure-eight coil. Sham stimulation was delivered with the parallel Magstim sham vacuum-cooled coil, which produces auditory signals and appears identical to an active coil but contains a mu-metal shield that diverts the majority of the magnetic flux such that a minimal (<3%) magnetic field is delivered to the cortex. All participants were told to expect that TMS sensations can differ during rTMS vs. RMT and were asked about side effects at the end of the visit. Participants, parents, study coordinator administering the TST, and TST video coders were masked to TMS group; the TMS operator was unmasked.

### 2.7 Analysis plan

Given the small sample size and single-subject experimental design, results were examined using descriptive statistics. *Post hoc* power analysis for between-group differences in TST change pre-post rTMS change confirmed that the study was not powered for formal inference and more appropriately examined using effect size estimation. (e.g., achieved power = 0.08–0.20 for two-sample, two-tailed *t*-test with α = 0.05). Therefore, Cohen’s *d* effect sizes were calculated to understand the magnitude of differences observed between the sham and active rTMS groups for change in tic frequency and premonitory urge intensity from pre- to post-rTMS in each TST condition. Interpretation of Cohen’s *d* values followed established conventions [small = 0.2, medium = 0.5, large ≥ 0.8; (Sullivan and Feinn, 2012)].

After examining TST tic frequency data pre-rTMS, we observed that 4 of the 14 participants (*n* = 3 active, *n* = 1 sham) demonstrated a strong baseline ability to voluntarily control tics, which we defined as a tic frequency of <2 tics/min during the pre-TMS Suppression conditions. In other words, these participants did not have much “room to improve” their tic suppressibility, resulting in a data floor effect (near zero tic frequencies pre and post-rTMS in SUP and SUP+R; none showed increased tic frequencies or urges post-rTMS). This ability to suppress to “near-zero” levels was consistent with previous TST research suggesting it to be present in approximately 20% of youth (Conelea et al., 2018). We conducted sub-analyses for tic frequency change in SUP and SUP+R excluding these “strong suppressors” to explore whether rTMS might improve suppression among those who have difficulty doing so.

There were no significant group-wise differences in urges or tic frequency in any TST conditions at baseline. There were no missing tic frequency data; two participants had missing average urge ratings for FT pre- and post-TMS. These participants were listwise deleted only for one analysis where this was the outcome variable.

## 3 Results by TST condition

**FIGURE 2:**
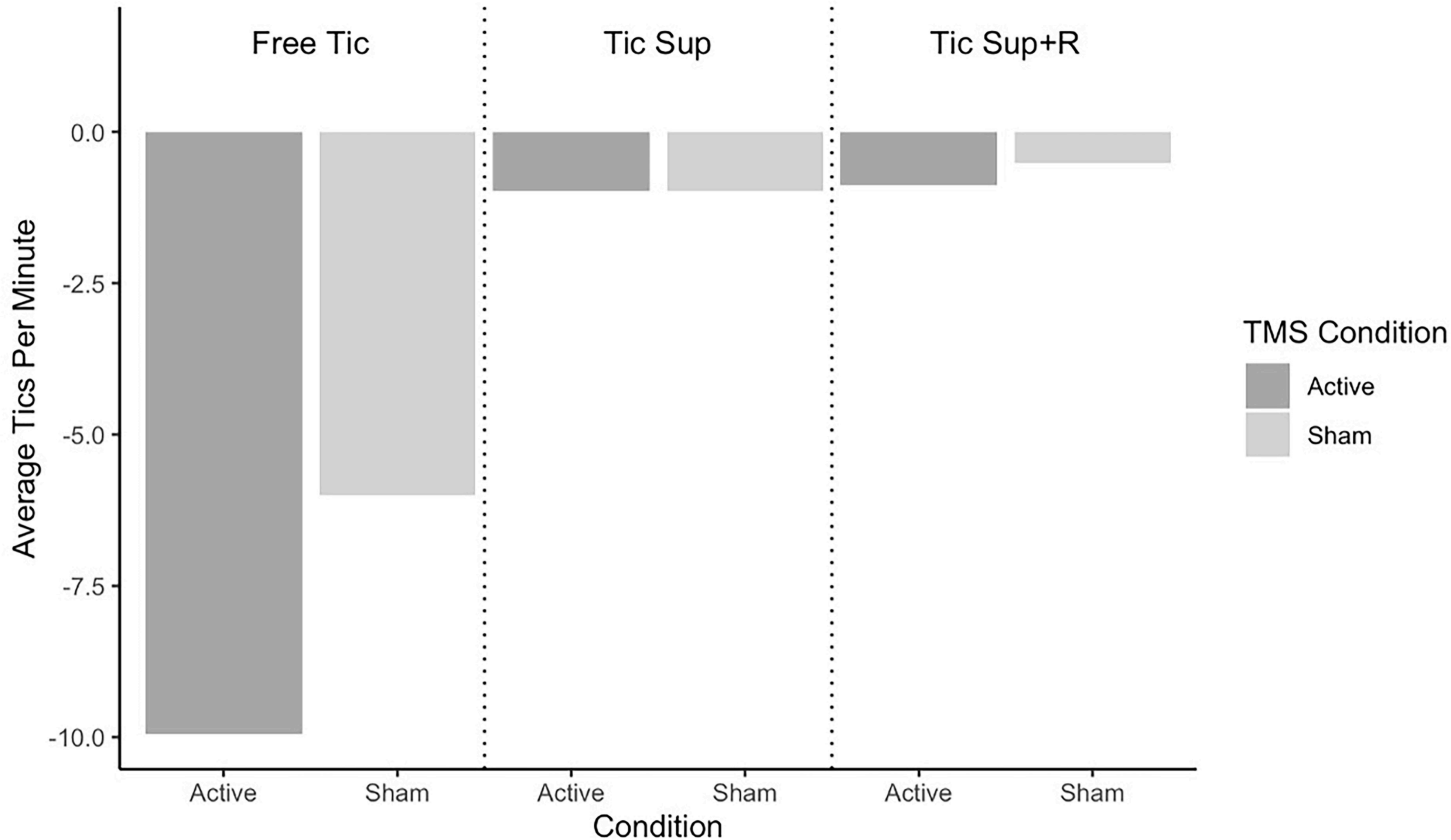
Full sample change pre-post rTMS in tics per minute by Tic Suppression Task (TST) and transcranial magnetic stimulation (TMS) condition.

**FIGURE 3:**
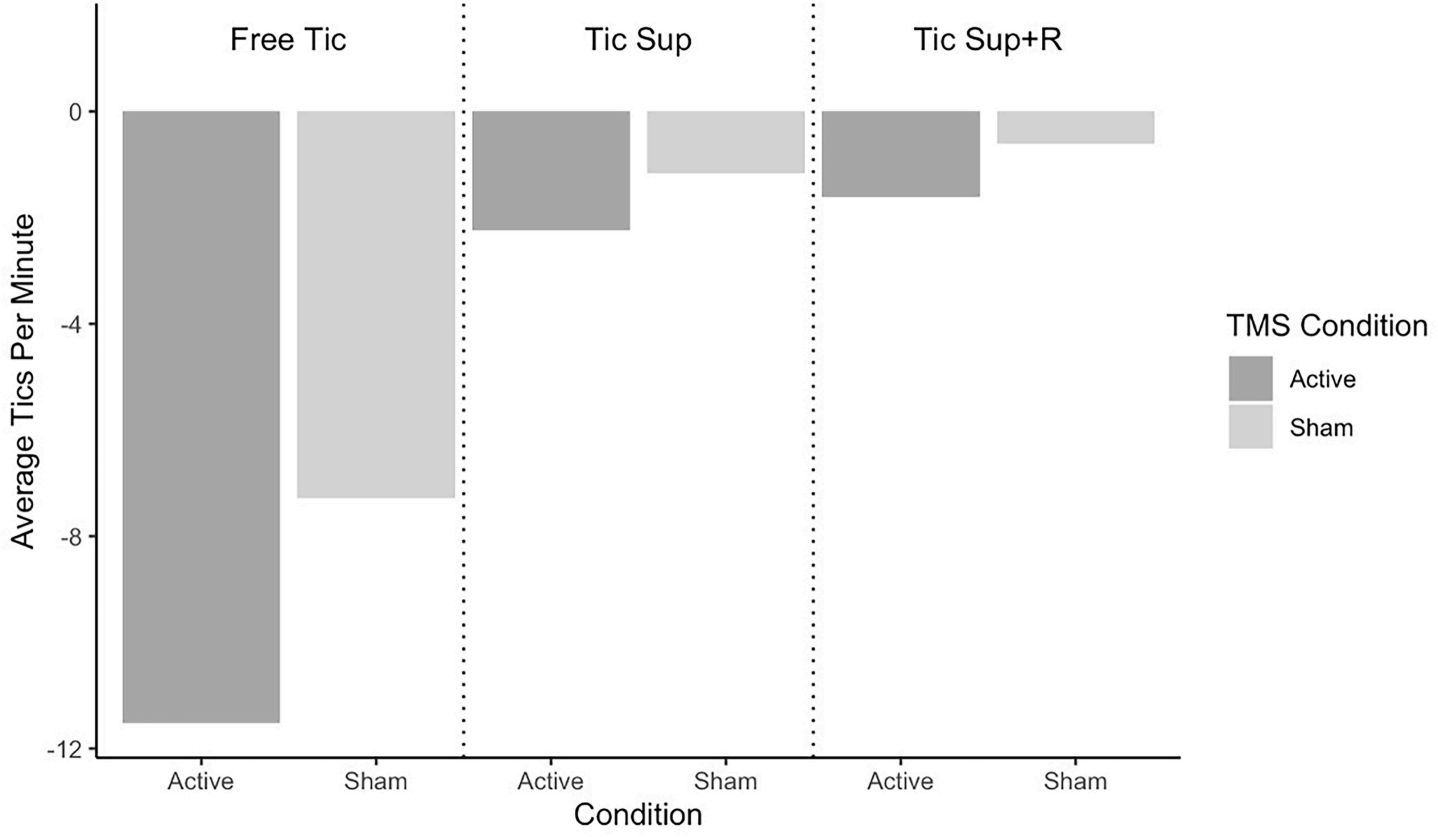
Non-suppressors change pre-post rTMS in tics per minute by Tic Suppression Task (TST) and transcranial magnetic stimulation (TMS) condition.

**FIGURE 4:**
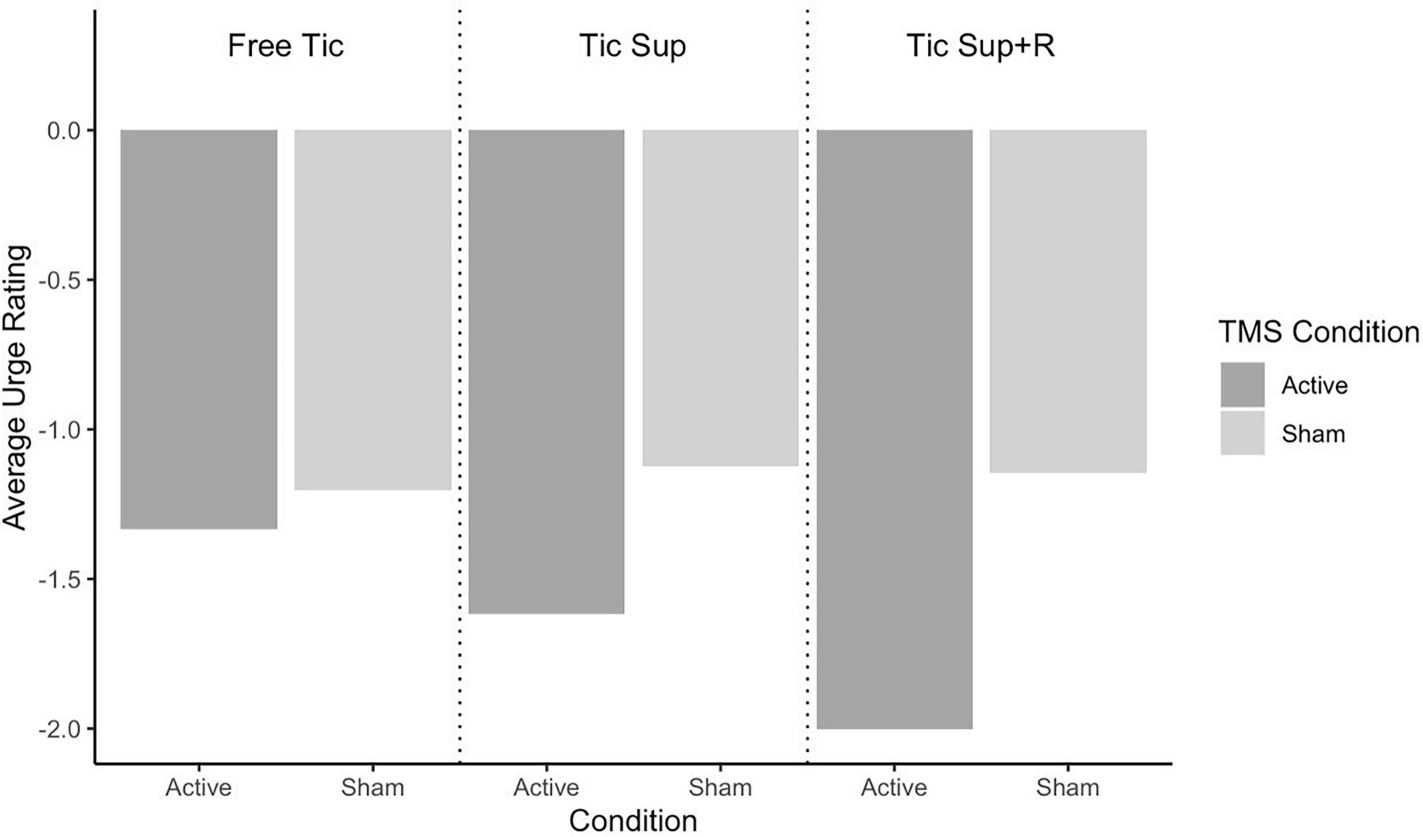
Full sample change pre-post rTMS in average urges by Tic Suppression Task (TST) and transcranial magnetic stimulation (TMS) condition.

**FIGURE 5:**
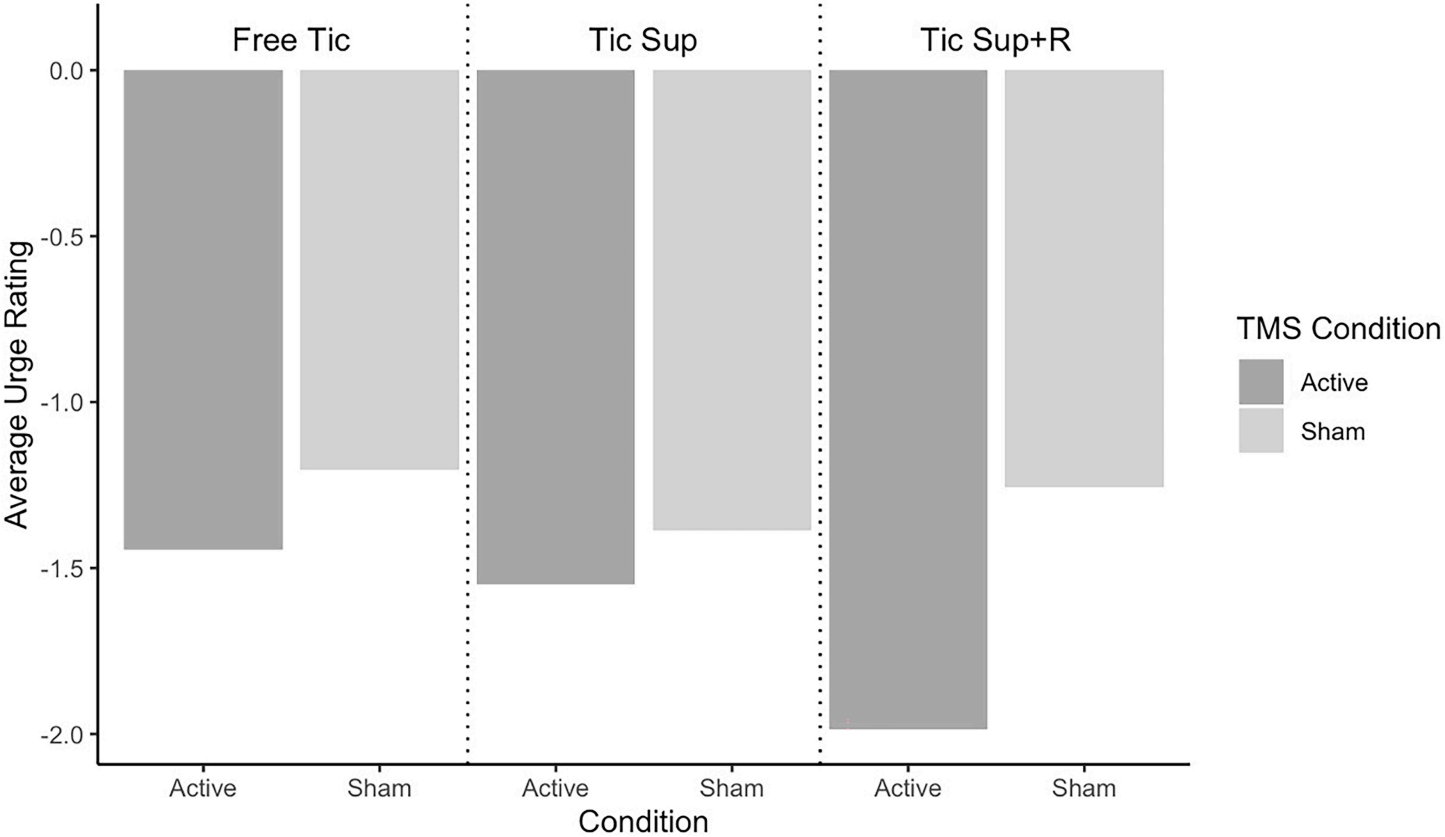
Non-suppressors change pre-post rTMS in average urges by Tic Suppression Task (TST) and transcranial magnetic stimulation (TMS) condition.

Average change in tic frequency by TST condition and rTMS assignment for the full sample is depicted in Figure 2 and for the sub analysis removing “strong suppressors” in Figure 3. Average change in premonitory urge intensity for the full sample is presented in Figure 4 and for the subsample in Figure 5.

**Free-to-Tic:** The active rTMS group showed a greater reduction in tic frequency in this condition (mean Δ = −9.9 tics/min, SD = 11.7) as compared to sham (mean Δ = −5.9 tics/min, SD = 11.5), with a small effect size of *d* = 0.34. We did not observe a group-wise difference in the change of premonitory urge ratings (sham: mean Δ = −1.20, SD = 1.71; active: mean Δ = −1.33, SD = 1.18; *d* = 0.09).

**Suppression:** In the full sample, tic frequencies following both sham and active rTMS did not notably change in this condition (sham: mean Δ = −0.97 tics/min, SD = 1.53; active: mean Δ = −0.97 tics/min, SD = 4.26; *d* = 0.0). When the participants classified as “strong suppressors” were removed, we observed greater reduction in tic frequency for those who received active rTMS (mean Δ = −2.24 tics/min, SD = 1.63; sham: mean Δ = −1.17 tics/min, SD = 1.63) with a small effect size of *d* = 0.26. Premonitory urge intensity decreased slightly more in active (mean Δ = −1.62, SD = 2.80) vs. sham rTMS (mean Δ = −1.12, SD = 2.23), with an effect size just under small (*d* = 0.19).

**Suppression+Reward:** In the full sample, tics were less frequent following active rTMS (mean Δ = −0.88 tics/min, SD = 1.88) vs. sham (mean Δ = −0.51 tics/min, SD = 0.68), small effect size of *d* = 0.24. Excluding the “strong suppressors,” we observed a stronger, medium-sized effect of active rTMS (mean Δ = −1.62 tics/min, SD = 2.28; sham: mean Δ = −0.61 tics/min, SD = 0.71), *d* = 0.63. Premonitory urge intensity during SUP+R decreased more in the active rTMS group, with a medium effect size (sham: mean Δ = −1.15, SD = 0.91; active: mean Δ = −2.00, SD = 2.01; *d* = 0.52).

## 4 Discussion

The aim of the current study was to probe the functional role of SMA in natural tic expression, voluntary tic control, and premonitory urge intensity using a single session of inhibitory 1Hz rTMS to acutely induce change in SMA function. TST administration immediately before and after active or sham rTMS enabled us to examine stimulation effects on these variables during periods of natural tic expression and attempted suppression. Results suggest that active rTMS led to reductions in tic frequency, reductions in premonitory urge intensity during suppression, and improved tic controllability. This overall pattern of results is consistent with prior literature pointing to SMA hyperactivation in TS.

Tic frequencies following active rTMS showed the most reduction during periods of natural tic expression (i.e., Free-to-Tic) and attempted suppression paired with contingent rewards (i.e., Suppression+Reward), albeit with small-to-medium effect sizes. These findings are convergent with basic research on SMA’s role in gating motor signals that travel to the primary motor cortex. SMA integrates information from frontal, proprioceptive, and cognitive areas to enable activation of an appropriate response and inhibition of inappropriate responses (Nachev et al., 2008). Importantly, this “activation/inhibition” process integrates information about reward expectancy signals (Campos et al., 2005) and prior reward learning history (Chen et al., 2010; Wardak, 2011). Given this, it makes sense that induction of “more normative” SMA activity would improve the brain’s overall ability to (a) inhibit tic signals, (b) activate alternative motor actions, and (c) withhold tics more robustly in the presence of suppression-contingent rewards. Future research should examine whether neural indices of SMA activity and/or connectivity are predictive of tic frequencies during the TST, as well as explore whether developmental changes in SMA may contribute to the observation that some youth tic less as they age (Hassan and Cavanna, 2012). From an intervention perspective, these data converge with prior studies pointing to SMA as a potentially beneficial target TS treatment and further suggest that pairing rTMS with training and practice of tic control strategies may facilitate better learning, particularly when including exposure to rewarding consequences.

Our secondary tic frequency analysis focused on participants who showed baseline difficulty controlling tics. In this subset, we observed a more robust effect of active rTMS on tic frequencies during both suppression conditions (Suppression, Suppression+Reward). This finding may be a byproduct of reducing the floor effect. Alternatively, it may suggest particular therapeutic potential for rTMS in youth with poor tic suppression ability. Notably, the current first-line treatment for tics, Comprehensive Behavioral Intervention for Tics [CBIT; (Woods et al., 2008)], is designed to improve tic control and thus relies to some extent on a pre-existing ability to suppress. A lack of this ability may be one reason for CBIT non-response, which is unfortunately the outcome for about 50% of youth (Piacentini et al., 2010). Indeed, prior research using the TST has shown that tic suppression ability is widely variable (Conelea et al., 2018) and includes a sizable proportion of youth (17%) who show no reduction or even tic worsening when attempting suppression. Clinical trials combining rTMS and CBIT are underway (Conelea et al., 2023; Kahl et al., 2021b), which is a logical next step given these findings. It will be particularly interesting to understand whether TST-measured tic suppressibility is a predictor of outcome in both CBIT alone and CBIT plus rTMS, as it may eventually be a useful tool for treatment selection.

We also examined rTMS-related effects on self-report ratings of premonitory urge intensity during TST conditions. Prior research implicating SMA hyperactivation in the premonitory urge experience (Cavanna et al., 2017) led us to hypothesize that active rTMS would reduce urge intensity. Interestingly, active rTMS did not impact urges during periods of natural tic expression, but it did lead to more urge reduction during both suppression conditions, particularly Suppression+Reward. In some ways, the suppression-specific effect is not entirely surprising. Urge intensity is typically rated lower during natural tic expression than suppression (Brandt et al., 2016; Capriotti et al., 2014), likely because urges are relieved very quickly by tics or bouts of tics (Wellen et al., 2024). Some have argued that suppression efforts are a prerequisite for urges, such that urge ratings reflect awareness of the effort involved in withholding tics (Jackson et al., 2011). Viewed this way, it is likely that any possible effect of rTMS on urges may be best detected if measured in a suppression context.

Our findings suggest that SMA plays a role in mediating perceived urge intensity, specifically during suppression. This is consistent with literature showing that SMA is a key node in the “urge-for-action” network that underlies both natural (e.g., yawning) and pathological physiological urges for action (Zouki et al., 2024). How stimulation impacted urge ratings is not entirely clear. Stimulation may have blunted premonitory sensations, particularly if it altered activity in the “action” cingulo-opercular network (D’Andrea et al., 2023) or SMA projections to primary motor or sensorimotor cortices. Alternatively, or in addition, stimulation may have impacted the perception or cognitive interpretation of premonitory sensations. SMA plays a role in adjusting effort intensity and motor force (Dai et al., 2001), and prior research has shown that inhibitory rTMS to SMA decreases the perception of physical effort during a grip force task (Zénon et al., 2015). Finally, given that tic frequency was lower after active rTMS, it is also possible that urges decreased as a byproduct of tic reduction (i.e., fewer opportunities to experience the urge). In this study, as in others, our ability to measure premonitory phenomena remains quite crude, making it difficult to dissociate these possibilities empirically. Future efforts should include multi-modal methods and more time-dense measurement [e.g., (Wellen et al., 2024)] to further clarify SMA’s role in the premonitory urge experience.

From a clinical perspective, it is meaningful that urges were perceived to be less uncomfortable during suppression after active rTMS. Premonitory urges in childhood uniquely predict greater impairment (Cavanna et al., 2012) and tic severity (Ricketts et al., 2022) later in life, and they can be more bothersome than tics themselves (Cohen and Leckman, 1992). “Urge intolerance,” the inability to tolerate urge-rated distress, predicts greater tic severity and tic impairment and may interfere with the ability to benefit from CBIT (Ramsey et al., 2021, 2022). Perceived urge intensity may therefore be a beneficial treatment target, and rTMS to SMA a viable method for directly targeting the urge experience.

Limitations of this study include the small sample size, which limited analyses to focus on effect sizes and prevented meaningful examination of potential moderators of rTMS effects. This includes variables related to pubertal stage and sex/gender differences, which will be important for future research to examine given known sex differences in TS prevalence and phenomenology (Garris and Quigg, 2021). Although anecdotal observations supported blinding adequacy, formal assessment of participant beliefs about the type of TMS received was not done and will be critical to assess in future trials. Finally, is important to emphasize that rTMS was delivered in a single session, and TST outcomes were examined when stimulation after-effects were likely to still be present (Ji et al., 2017). It is unclear if the pattern of observed changes would be similar following a “treatment grade” rTMS protocol (i.e., repeated stimulation sessions meant to induce durable neural change).

The TMS targeting protocol we used accounted for individual differences in brain anatomy and SMA function but still relied on subjective interpretation for final target selection. It is possible that stimulation may have engaged different functional networks or subregions of SMA across participants. Significant advances in quantitative, automated targeting methods have been made since this study was designed, enabling better focality and consideration of individual neural network topography, distribution and intensity of the TMS-induced electric-field, and stimulation “dose” delivered “on target” (Elbau et al., 2023; Lynch et al., 2022). These types of targeting methods should be used in future studies using TMS to probe brain function, as it increases confidence around independent variable integrity.

Finally, we acknowledge that the focus on SMA as a region throughout this report may obscure the importance of its role as a connected hub within cortico-striatal circuits. rTMS, though delivered focally, has been shown to induce downstream effects in brain areas that are functionally and structurally connected, including subcortical regions (Oathes et al., 2021). Since this study did not include any measures of neural function alongside stimulation, it is not possible to empirically confirm the neural mechanisms by which stimulation may have ultimately impacted behavioral outcomes. Replication of this study with newer technologies to enable “online” measurement of brain activity alongside the TST, such as EEG-TMS, will be valuable.

## Data Availability

The dataset presented in this article is not readily available because it contains protected health information. Requests to access the dataset should be directed to the corresponding author cconelea@umn.edu.
